# The Application of a Contact Lens Sensor in Detecting 24-Hour Intraocular Pressure-Related Patterns

**DOI:** 10.1155/2016/4727423

**Published:** 2016-07-25

**Authors:** Sarah C. Xu, Angela C. Gauthier, Ji Liu

**Affiliations:** ^1^Department of Ophthalmology and Visual Science, Yale University School of Medicine, New Haven, CT 06510, USA; ^2^Department of Ophthalmology, Mayo Clinic, Rochester, MN 55905, USA

## Abstract

Glaucoma is one of the leading causes of blindness worldwide. Recent studies suggest that intraocular pressure (IOP) fluctuations, peaks, and rhythm are important factors in disease advancement. Yet, current glaucoma management remains hinged on single IOP measurements during clinic hours. To overcome this limitation, 24-hour IOP monitoring devices have been employed and include self-tonometry, permanent IOP, and temporary IOP monitoring. This review discusses each IOP measuring strategy and focuses on the recently FDA-approved contact lens sensor (CLS). The CLS records IOP-related ocular patterns for 24 hours continuously. Using the CLS, IOP-related parameters have been found to be associated with the rate of visual field progression in primary open-angle glaucoma, disease progression in primary angle-closure glaucoma, and various clinical variables in ocular hypertension. The CLS has been used to quantify blink rate and limbal strain and measure the circadian rhythm in a variety of disease states including normal-tension glaucoma and thyroid eye disease. The effects of various IOP-lowering interventions were also characterized using the CLS. CLS provides a unique, safe, and well-tolerated way to study IOP-related patterns in a wide range of disease states. IOP-related patterns may help identify patients most at risk for disease progression and assist with the development of tailored treatments.

## 1. Introduction

Glaucoma is a progressive optic neuropathy characterized by visual field loss and structural changes to the optic nerve. It is one of the leading causes of blindness worldwide along with cataracts and age-related macular degeneration [1–4]. Population-based studies on glaucoma have reported a prevalence rate in the range of 1.5–4.2% throughout the world with developing nations carrying a greater burden of bilateral blindness from glaucoma [5–9]. It has been estimated that 80 million people will be diagnosed with glaucoma and 11.2 million will be blinded by the disease by 2020 [10]. This troubling prediction is mainly due to aging and a growing world population.

Although it is no longer part of the modern definition of glaucoma, uncontrolled intraocular pressure (IOP) remains the single most important risk factor for glaucoma development and progression. Recent studies suggest that IOP fluctuations and peaks are associated with disease advancement. Nouri-Mahdavi et al. found that large IOP fluctuations and age carry significant odds ratios for visual field loss in the Advanced Glaucoma Intervention Study [11]. Similarly, De Moraes et al. found that IOP peak and fluctuation were associated with an increased risk for visual field progression in the Glaucoma Progression Study. Peak IOP was also found to be a significant risk factor in the multivariable model for visual field loss (OR = 1.13), along with thinner central corneal thickness, disc hemorrhage, and beta-zone parapapillary atrophy [12]. The association between IOP peaks and disease progression highlights the importance of studying IOP parameters during a 24-hour period.

Clinicians have realized the limitations in our current IOP measurement method for quite a few years. In most clinical practices in the United States, IOP is measured once during a patient's clinic visit using Goldmann applanation tonometry. The Goldmann tonometer measures the force needed to deform the spherical cornea to a standardized diameter based on the Imbert-Fick law [13]. The office measurements provide only cross-sectional views of patients' fluctuating IOP. Additionally, IOP measurements obtained by the Goldmann tonometer are operator dependent and influenced by patient position and ocular factors, such as astigmatism and central corneal thickness [14–16]. As a result, clinical details such as short-term IOP fluctuations within a 24-hour period and other IOP parameters remain hidden by our current IOP measurements using the Goldmann tonometer. This review discusses innovative approaches to measuring IOP during a 24-hour period, particularly the use of a contact lens sensor (CLS).

## 2. Strategies in 24-Hour Pressure Monitoring

Given the limitations of our current IOP measurement method, researchers and clinicians have devised three main strategies of 24-hour IOP monitoring: self-tonometry by the patient, permanent IOP monitoring, and temporary IOP monitoring [17]. In terms of self-tonometry, the Pulsair-Keeler was one device invented for patients to measure IOP at home to supplement the office visit IOP. The Pulsair-Keeler tonometer is a hand-held noncontact tonometer that measured IOP by indenting the cornea using a puff of air from the machine held 2 cm from the cornea [17]. However, self-tonometry was only achieved 75% of the time due to difficulty with usage. Of the measurements obtained, only 73% were accurate within 1 mmHg of measurements from the Goldmann tonometer [18]. Consequently, this device was not adopted for clinical use. Other self-tonometers trialed include the Proview Eye Pressure Monitor (also known as phosphine self-tonometry) and Ocuton S, both of which were based on the same principle of applanation, like the Goldmann tonometer. Despite training, 41% to 47% of patients could not perform self-tonometry with Ocuton S [19, 20]. Numerous studies have showed limited correlation in the IOP readings from Proview and Goldmann, thus questioning the effectiveness of the Proview tonometer [8, 21, 22]. A self-tonometer offering good correlation with the Goldmann is the iCare rebound tonometer [23]. It is a hand-held device that measures IOP through deceleration of a magnetized probe after it rebounds from the cornea. Out of 76 subjects, 74% were able to correctly preform the self-tonometry. Central corneal thickness below 500 *µ*m or above 600 *µ*m was correlated with greater difference between Goldmann and iCare [23]. Despite the various self-tonometers available, these devices do not allow continuous measurement of 24-hour IOP patterns during undisturbed sleep. Furthermore, they are limited by challenges of usability and IOP recording accuracy.

Implantable, permanent IOP sensors provide the second strategy in IOP monitoring. Chen et al. in 2006 designed an intraocular sensor that can be fixed to the iris. The principle behind this device is the use of a Bourdon tube that mechanically deforms depending on the IOP [24]. Another permanent sensor is designed as an artificial intraocular lens with an embedded sensor, which detects IOP-induced stress and causes a change in the resistance of the circuit [25–27]. The advantage of these devices is that they would allow continuous, direct measurement of true IOP, particularly during undisturbed sleep. However, these devices require surgical implantation, which is associated with risks including infection, bleeding, inflammation, and device failure. To date, none of the designs have advanced to the human trials phase [8].

Finally, temporary IOP devices have the most potential to be widely used given that no surgical intervention is necessary and the sensor is easily reversible. Leonardi et al. designed and tested the first contact lens sensor [28]. A soft contact lens is embedded with strain gauges to measure the circumferential changes at the corneoscleral junction. The principle behind the device is that a change in IOP of 1 mmHg is associated with a 3 *µ*m change in the corneal curvature radius [29, 30]. The electrical output (mV Eq) change from the sensor has been shown to significantly correlate with IOP changes. Leonardi et al. showed in 2009 that, in enucleated pig eyes, electrical output from the contact lens sensor had good sensitivity and correlation with IOP in the range of 17 to 29 mmHg [31]. Since then, the device has also shown good sensitivity and tolerability in human clinical trials [32].

## 3. What Is the Triggerfish® 24-Hour Contact Lens Sensor?

**Figure 1:**
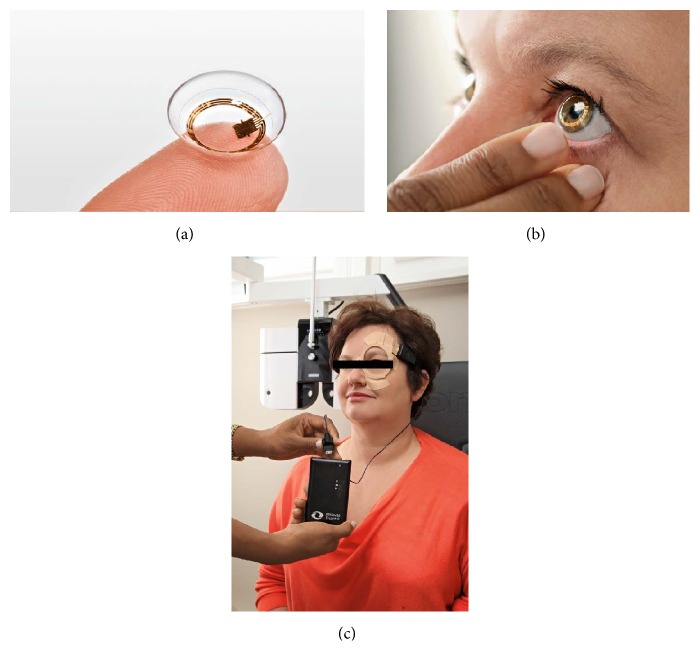
(a) SENSIMED Triggerfish contact lens sensor. (b) The contact lens sensor worn on the eye. (c) The device system consists of a wireless contact lens sensor, a periorbital antenna, data cable, and a recorder. Images are reproduced from SENSIMED Triggerfish with permission from the company.

The Triggerfish is a soft contact lens sensor tested by Leonardi et al. [28]. This CLS provides a minimally invasive method to record IOP-related ocular patterns for 24 hours continuously [31]. The CLS consists of resistive platinum-titanium strain gauges embedded in the soft silicon contact lens (Figure 1(a)). Depending on the patient's cornea steepness, three different sizes of the contact lens are available to allow for an appropriate fit. The device is placed on the cornea as shown in Figure 1(b). The CLS sends electrical signals via Bluetooth® to an antenna which is patched periorbitally, before transmitting and storing the information in a recorder (Figure 1(c)). Patients wear the device for 24 hours with minimal restriction in activities. The 24-hour IOP-related ocular pattern data are retrieved after connecting the recorder to a computer. The CLS produces electric output in millivolts (mV). When the output is trended over time, the resulting graph is termed IOP-related patterns or profiles. The output has been hypothesized to measure a composite of IOP, anterior volume change, and ocular biomechanical properties [32–34]. Thus far, this CLS has been used safely and effectively in healthy subjects, patients with open-angle glaucoma, and patients with angle-closure glaucoma [32, 34–36]. It has also been used to show the effect of IOP-lowering interventions during a 24-hour period, particularly the nocturnal period [37].

## 4. Safety, Reproducibility, and Accuracy

The most common adverse events include conjunctival hyperemia, blurred vision, and superficial punctate keratitis as reported by Mansouri et al. [38]. In the initial clinical study, one case of corneal erosion in a patient with severe dry eye disease and four cases of superficial punctate keratitis were reported in a cohort of 15 patients, all of which resolved within 24 hours [39]. In our study that fitted 17 ocular hypertensive patients with the CLS unilaterally, all patients experienced self-resolving, mild conjunctival hyperemia [40]. In a study that examined the safety and tolerability of the CLS in patients with glaucoma, Mansouri et al. found that 82% of participants developed blurred vision, 80% conjunctival hyperemia, and 15% superficial punctate keratitis after CLS use [38].

The CLS has been found to be an accurate and reproducible method to characterize the nyctohemeral IOP rhythm. In a cross-sectional study of 12 young healthy volunteers, Mottet et al. found fair-to-good agreement (i.e., 95% confidence interval) between CLS measurements in separate recording sessions [32]. By comparing output from the CLS to output from a noncontact tonometry (Pulsair-Keeler) in the fellow eye, Mottet et al. showed that the CLS tended to overestimate the high IOP changes in comparison with the noncontact tonometry [32]. The authors postulated that the difference is likely a result of the higher frequency of data acquisition by the CLS, thus reducing the influence of outliers.

24-hour CLS use has been shown to cause overnight corneal swelling, particularly in the central and mid-peripheral zones. However, Freiberg et al. did not find a correlation between pre- and postcentral corneal thickness and CLS output difference. These data suggest continuous CLS output is not significantly affected by differences in corneal thickness that occurred during overnight CLS wear [41].

Furthermore, output from the CLS has been used to accurately identify sleep and wake periods. Gisler et al. derived an algorithm to process the output and was able to identify sleep periods with 95% accuracy [42]. The authors also quantified eye blinks using CLS output and validated the measurements with simultaneous video recording in a subset of the participants.

## 5. Clinical Applications

In glaucoma patients, output from the CLS has been used to predict the rate of visual field loss. In a prospective cross-sectional study, 34 treated, open-angle glaucoma patients with at least 8 recorded 24-2 Humphrey visual field tests over 2 years wore the CLS for 24 hours [43]. Based on visual field tests, the patients were divided into “fast progressors” and “slow progressors.” Various CLS parameters were measured, including the number of large peaks (excludes smaller peak artifacts) and the mean peak ratio (a higher mean peak height to time-to-peak indicates quick spikes in mV equivalents). “Fast progressors” tended to be predicted by the number of long peaks and the mean peak ratio. This indicates that CLS may be used to identify patients more likely to advance quickly in their disease so they can be treated more aggressively. A summary of this and studies below is provided in Table 1.

Glaucoma patients may be at higher risk of eye damage during sleep, especially if they lie face down, which can increase pressure on the eye. CLS has been used to investigate this risk in a study of 22 glaucoma patients and 11 age-matched controls [36]. First, the Tono-Pen was used to help determine that the glaucoma and control subjects' baseline IOPs while sitting and lying were similar. Then, the participants wore the CLS for approximately 3 hours while alternating positions of sitting, lying supine, and lying face down. Subjects with glaucoma experienced a sustained increase in CLS values while lying face down, but control subjects did not. This rise was equivalent to a mean IOP increase of 2.5 mmHg. Future efforts may be directed at identifying which glaucoma patients are most susceptible to this increased strain and developing therapies to mitigate it.

CLS has also been used to monitor the IOP-related profile in normal-tension glaucoma patients, which may help elucidate why they experience visual field loss seemingly without elevations in IOP. In one study, 18 subjects wore the CLS for 24 hours and data were collected detailing variability from the mean and the number of peaks and troughs that occurred during the day and night [44]. It found that there was 48.9% less variability and 54.7% less peaks during the night compared to the day. This may be due to less eye and body movements at night. The study also discovered that going to sleep was associated with a higher rate of increase in the IOP-related pattern than the decrease in such pattern associated with waking up. The authors postulated that the difference in IOP-related pattern change may be due to IOP increase from postural change. Further research aided by CLS may eventually help optimize treatment timing for each patient based on his/her IOP-related pattern throughout the day.

In primary angle-closure glaucoma, CLS has been used to study how differences in IOP fluctuations are associated with rates of disease progression. Tan et al. had 25 patients with primary angle-closure glaucoma wear the CLS for 24 hours, while they went about their daily activities [45]. The patients were classified as having “progressive” or “stable” glaucoma by measuring mean deviation, visual field index, and retinal nerve fiber layer thickness changes every 6 months. Progressive patients as defined by a significant change in the mean deviation had significantly different gradients (1st derivative) of the IOP fluctuation curve during the hours of 10:00 pm to 11:00 pm and 7:00 am to 8:00 am compared to stable patients. They also had significant differences in the curvature (2nd derivative) of the IOP curve between the hours of 11:00 pm and 12:00 am, as well as 8:00 am and 9:00 am. Significant differences were found between progressive and stable patients using the visual field index and retinal nerve fiber layer thickness criteria during several other hours as well. Overall, the CLS helped illustrate that patients with progressive glaucoma experienced larger variations in IOP fluctuation than the stable group during bedtime and waking hours.

The use of CLS is not limited to glaucoma patients; it has also been used to study patients with ocular hypertension. CLS monitoring in 17 untreated ocular hypertension patients revealed that patients who had poorer visual field mean deviation had steeper sleep to wake slopes [40]. In addition, a higher number of peaks within the 24 hours of monitoring were associated with greater fluctuations in IOP between office visits. Similar to glaucoma patients, ocular hypertension patients with worse disease may be more easily identified by CLS IOP-related patterns.

CLS may also be used to help patients with thyroid eye disease. Grave's Disease often involves ocular manifestations such as proptosis and diplopia due to mucopolysaccharide and lymphocyte infiltrates into the extraocular muscles. These infiltrates apply pressure to the globe, which may increase IOP. Goldmann applanation tonometry may produce inaccurate IOP readings because of the increased force required to pull the eye against the fibrotic and constrained extraocular muscles [46]. However, CLS can provide a more accurate way to follow IOP in these patients, as long as it is safe and well tolerated. A prospective study of 10 patients with thyroid eye disease used the CLS to monitor ambulatory IOP in the patients' more proptotic eye for 24 hours [46]. The study found that CLS was very well tolerated, achieving a score of 1.5 ± 0.7 on a scale of 0 (no discomfort) to 10 (very severe discomfort). In addition, it was deemed safe, as the most frequent adverse effects were only mild blurred vision (50% of patients), mild hyperemia of the bulbar and palpebral conjunctiva (100% of patients), and superficial punctate keratitis (20% of patients), all of which resolved after CLS removal. Fifty percent of the patients experienced nocturnal acrophase, with the peak occurring at 6:30 am. The timing of acrophase differs from glaucoma patients, who typically experience a peak between 1:00 am and 3:00 am [33]. This indicates that patients with thyroid eye disease may have a unique circadian IOP rhythm compared to those with glaucoma.

## 6. Effect of Interventions during a 24-Hour Period

The CLS was used to study the circadian IOP-related effects of ocular hypotensive medications. Mansouri et al. found in a cohort of 23 patients with primary open-angle glaucoma that prostaglandin analogues uniquely flattened the IOP-related increase during the wake-to-sleep period [37]. All patients initially underwent a four-week wash-out and then were fitted with the CLS for baseline measurements. Patients were randomly assigned to use bimatoprost 0.01% nightly, brimonidine 0.1% twice daily, brinzolamide 1% twice daily, or timolol 0.25% nightly for 1 month prior to a second CLS fitting. Patients treated with bimatoprost had a significant decrease in the slope during wake-to-sleep period, something which other treatment groups did not experience. This finding adds to the growing literature that prostaglandin analogues alter the nocturnal IOP-related pattern. A case series involving five normal-tension glaucoma patients showed flattening of the nocturnal IOP increase in three of the patients after introduction of a mixed group of medications, including prostaglandin analogue eye drops [35]. Another case study reported the IOP-related pattern change after treatment with prostaglandin-pilocarpine combination [48]. The patient presented with ocular pain after ab interno trabeculotomy (Trabectome*™*) surgery. Through the use of CLS, a significantly lower, nocturnal IOP-related pattern was associated with patient's improvement in symptoms.

The 24-hour effect of selective laser trabeculoplasty (SLT) has been examined in patients with normal-tension glaucoma using the CLS. Eighteen patients were fitted with CLS before and 1 month after SLT. The IOP-related pattern amplitude was reduced in patients with a successful SLT treatment (>20% in IOP reduction by Goldmann applanation). In contrast, the amplitude increased in patients with nonsuccessful SLT treatment [47]. Higher diurnal, local IOP-related variability and flatter sleep-to-wake slope were observed after SLT in patients with nonsuccessful treatment. This CLS study revealed the association between SLT treatment success and amplitude of IOP-related patterns.

## 7. Advantages and Limitations

Recently, the Triggerfish has gained the U.S. Food and Drug Administration (FDA) approval for its CLS application. It is currently the only FDA-approved, nonimplantable device for continuous 24-hour IOP-related pattern measurements. The CLS is indicated to detect the peak patterns of variation in intraocular pressure over a maximum period of 24 hours to identify the window of time to measure intraocular pressure by conventional clinical methods [49]. In addition to helping determine the most critical time of day for clinicians to measure a patient's IOP, parameters calculated using CLS output enhance our understanding of medication effects and identify patients with higher risk of disease progression.

As summarized above, CLS is a revolutionary tool in ophthalmology, especially in the glaucoma field, but the current cost of the device can be an obstacle for its wide use. Although every glaucoma patient may benefit from CLS for disease management, this device will be most helpful to those with faster disease progression or those who are less responsive to IOP-lowering therapy by the current standard. For most patients, glaucoma is a slow, progressive optic nerve disease. However, some patients may show worrisome faster visual function loss than average patients, even if their IOP seems adequately reduced during office visits. The CLS recording may help identify the ocular patterns of these patients and provide guidance to administer medicine more precisely to suppress IOP outside office hours. This could completely control intraocular pressure in this group of patients and also decrease the chance of exposing them to more aggressive and/or more invasive therapy. Similarly, the CLS can help determine the effectiveness of an IOP-lowering medicine for a patient before switching or escalating therapy, especially for those patients with low pressure glaucoma. In addition, the application of CLS on ocular hypertensive patients may help identify those who are more likely to develop glaucoma. This can help many patients in this group avoid unnecessary therapy, as studies showed ocular hypertension might not lead to glaucoma in every patient [50]. Therefore, as an approach to deliver precision medicine, the CLS may potentially decrease the total cost of glaucoma management and treatment risks.

One major limitation of the CLS is that its output cannot be converted to millimeter mercury (mmHg) to allow for direct clinical interpretation. However, because the ocular pattern recorded by CLS is highly correlated to the IOP rhythm, the readings obtained from the CLS can be used to guide clinicians to determine the critical time for IOP measurement during the 24-hour period, as stated by FDA [48]. In addition, current studies indicate that the IOP-related CLS profile itself can be used as an ocular perimeter for individualized glaucoma management. More studies are needed to determine which biomechanical properties determine the CLS output and whether the output is associated with corneoscleral properties, such as corneal hysteresis.

## 8. Conclusions

Using the CLS, IOP-related parameters have been found to be associated with the rate of visual field progression in primary open-angle glaucoma, disease progression in primary angle-closure glaucoma, and various clinical variables in ocular hypertension. The CLS has been used to quantify blink rate and limbal strain and measure the circadian rhythm in a variety of disease states including normal-tension glaucoma and thyroid eye disease. The effects of various IOP-lowering interventions were also characterized using the CLS. CLS provides a unique, safe, and well-tolerated way to study IOP-related patterns in many conditions. With its recent clearance from the FDA, wider usage of CLS may lead to a change of current practice patterns in glaucoma. IOP-related patterns may help identify patients most at risk for disease progression and assist with the development of tailored treatments.

## Figures and Tables

**Table 1:** Summary of studies using the CLS in the discovery of risk factors, characterization of circadian rhythm, and investigation of effects of IOP-lowering interventions.

Study	Study design	*n*	Mean age (yr)	Disease	Outcome measures	Main findings
De Moraes et al. [43]	Cross-sectional	34	66.8	Primary open-angle glaucoma	Rates of visual field change and CLS parameters	Number of long peaks and mean peak ratio were best predictors of faster progression

Flatau et al. [36]	Nonrandomized comparative trial	33	62.6	Glaucoma	Changes in limbal strain and sleeping head position	Limbal strain increased in glaucoma eyes with face down position, particularly those eyes with progressive visual field loss

Lee et al. [44]	Prospective cohort	18	65.1	Normal-tension glaucoma (NTG)	IOP-related pattern in nocturnal and diurnal periods	NTG patients have IOP-related pattern unique to diurnal/nocturnal period: greater diurnal variability and fewer nocturnal peaks

Tan et al. [45]	Nonrandomized comparative trial	25	69.1	Primary angle-closure glaucoma	Glaucoma progression and CLS parameters	Patients with progressive disease differ from stable patients in gradients of IOP fluctuation curve during specific time periods

Xu et al. [40]	Nonrandomized comparative trial	17	59	Ocular hypertension	Baseline clinical factors and CLS parameters	Poorer visual field mean deviation associated with steeper sleep to wake slopes; higher number of peaks associated with greater IOP fluctuations between office visits

Parekh et al. [46]	Prospective nonrandomized trial	10	61.8	Thyroid eye disease	Safety, tolerability, and IOP-related pattern	Well-tolerated and safe CLS; 50% of patients experience nocturnal acrophase with peak at 6:30 am

Mansouri et al. [37]	Randomized controlled trial	23	63.8	Primary open-angle glaucoma	Effect of different groups of medications	Bimatoprost uniquely decreased the IOP-related slope during the wake-to-sleep period

Pajic et al. [35]	Nonrandomized trial	5	62	Normal-tension Glaucoma	Effect of different groups of medications	Treatment with latanoprost, travoprost, and/or dorzolamide altered the IOP-related slope during the wake-to-sleep period

Lee et al. [47]	Prospective cohort	18	65.1	Normal-tension Glaucoma	Effect of SLT	Amplitude of CLS fitted curve was reduced by 24.6% in successful treatment group

Mansouri et al. [48]	Case report	1	59	Primary open-angle glaucoma	Effect of prostaglandin-pilocarpine	Prostaglandin-pilocarpine reduced nighttime IOP peaks and relieved patient's symptoms
